# IFITM3 affects the level of antibody response after influenza vaccination

**DOI:** 10.1080/22221751.2020.1756696

**Published:** 2020-05-18

**Authors:** Na Lei, Yan Li, Qiang Sun, Jian Lu, Jianfang Zhou, Zi Li, Liqi Liu, Junfeng Guo, Kun Qin, Haibin Wang, Jianhong Zhao, Chong Li, Lingli Sun, Dayan Wang, Zhendong Zhao, Yuelong Shu

**Affiliations:** a National Institute for Viral Disease Control and Prevention, Chinese Center for Disease Prevention and Control, Beijing, People’s Republic of China; b Chaoyang District Center for Disease Prevention and Control, Beijing, People’s Republic of China; c School of Public Health (Shenzhen), Sun Yat-sen University, Guangdong, People’s Republic of China; d NHC Key Laboratory of Systems Biology of Pathogens, Institute of Pathogen Biology, Chinese Academy of Medical Sciences and Peking Union Medical College, Beijing, People’s Republic of China

**Keywords:** Interferon-induced transmembrane protein 3, IFITM3, SNP rs12252, influenza virus, trivalent inactivated vaccine, immune response

## Abstract

Interferon-induced transmembrane protein 3 (IFITM3) as an antiviral factor can inhibit replication of several viruses including influenza virus. A single-nucleotide polymorphism rs12252-C of *IFITM3* results in a truncated IFITM3 protein lacking its first 21 amino acids, which is much higher in the Han Chinese population and associated with severe illness in adults infected with pandemic influenza H1N1/09 virus. To investigate if IFITM3 or *IFITM3* rs12252-C could affect the antibody response after influenza vaccination, we detected the haemagglutination inhibition (HI) of 171 healthy young adult volunteers (*IFITM3* rs12252-C/C, C/T, T/T carriers) and in an IFITM3-deletion mouse model (*Ifitm3^-/-^*) after trivalent inactivated vaccine (TIV) immunization. Seroconversion rates for H1N1, H3N2 and B viruses in *IFITM3* rs12252-C/C genotype carriers was lower compared with C/T and T/T donors**.** Significantly lower levels of specific antibodies to H1N1, H3N2 and B viruses and total IgG were observed in *Ifitm3^-/-^* mice. Correspondingly, the numbers of splenic germinal centre (GC) B cells, plasma cells, TIV-specific IgG^+^ antibody secreting cells and T follicular helper cells in *Ifitm3^-/-^* mice were lower compared with wild type mice. However, the number of memory B cells was higher in *Ifitm3^-/-^* mice at day 7 after booster. The HI level of *Ifitm3^-/-^* mice remained lower than WT mice after third vaccination. Moreover, the transcriptional network regulating GC B cell and plasma cell differentiation was abnormal in *Ifitm3^-/-^* mice. Our results indicate that IFITM3 deletion attenuated the antibody response. The mechanism of influenza-IFITM3 interactions affecting the antibody response requires further investigation.

## Introduction

Influenza continues to threaten global public health and causes major morbidity and mortality in humans during seasonal epidemics, occasional pandemics, and zoonotic outbreaks [1]. Influenza A virus (IAV) has evolved various strategies to evade host defenses and acquire antiviral drug resistance [2]. The host produces a variety of factors that are able to fight Influenza virus infection through various mechanisms. Interferon (IFN)-induced transmembrane protein (IFITM) 3 is upregulated upon stimulation of cells by type I and II IFNs [3]. IFITM3 is a 15-kDa membrane-associated protein that localizes to late endosomes and lysosomes and inhibits viral entry into the host cell cytoplasm by impeding the formation of the virus fusion pore [4,5]. IFITM3 was identified as a host antiviral factor following RNA interference genomic screening for host factors involved in influenza virus infection during the pandemic influenza H1N1/09 virus outbreak [6]. IFITM3 was found to be one of the most potent antiviral factors in restricting influenza virus infection, as IFITM3 knockout mice displayed enhanced morbidity and mortality associated with pandemic influenza H1N1/09 virus infection [7]. Several distinct studies have revealed that IFITM3 plays a role in immune function [8]. IFITM3 was constitutively expressed in mouse lung-resident memory CD8^+^ T cells after challenge with influenza virus, enabling the mice to withstand viral infection during a secondary challenge and effecting quick protection at the site of viral entry [9]. Additionally, respiratory dendritic cells up-regulate the expression of IFITM3 in response to influenza virus infection [10].

An important single nucleotide polymorphism (SNP) rs12252-C of *IFITM3* is a splice variant wherein the majority allele, T, is substituted with a C and then encodes an *IFITM3* isoform (△21 IFITM3) lacking 21 amino acids at the amino terminus [11]. In humans, the SNP rs12252-C allele of *IFITM3* was linked to severe illness in adults during H1N1/09 virus infections, as well as following infection with the novel H7N9 virus [11,12]. The underlying mechanisms remain unclear, but it is known that the rs12252-C allele reduces the restriction on virus replication [11,13]. Research has shown that deleting the first amino-terminal 21-amino-acid or mutating 20-YEML-23 result in relocation of IFITM3 from the endosomal compartments to the plasma membrane, thus losing influenza-inhibitory action [13,14]. Moreover, a recent study found that rs12252-C/C donors had a significantly higher level of antibodies to H1N1/09 virus in comparison with rs12252-T/T donors before vaccination with the trivalent inactivated vaccine (TIV) and at 1 year post TIV [15].

Vaccination is the most effective method to prevent influenza virus infection. Growing evidence suggests that host factors including host genetics, the hormonal milieu, and gut microbiota play important roles as modifiers of influenza virus vaccine efficacy [16]. We are curious to know whether there is an association between IFITM3 and antibody response after TIV immunization. In this study, we recruited 171 healthy young adult volunteers who received TIV and observed the seroconversion rates (SCRs) for H1N1, H3N2 and B viruses. SCRs were found to be lower in donors with *IFITM3* rs12252-C/C genotype compared with rs12252-C/T or T/T genotype. We also found a lower level antibody after TIV booster in *Ifitm3^-/-^* mice. Further analysis of germinal centre (GC) B cell and plasma cell indicated a similar reduction in *Ifitm3^-/-^* mice. These findings suggest IFITM may affect the antibody response after influenza vaccination.

## Materials and methods

### Study cohort and ethics

A total of 212 healthy young adult volunteers (age from 18 to 59 years) from the Xinjiang autonomous region and Yunnan province were enrolled in the study from 2009 to 2015. People who infected with influenza virus within 3 months and self-reported influenza vaccination previously were excluded. All volunteers were injected once with licensed seasonal TIV in a volume of 0.5 mL, and whole-blood specimens were collected at day 0 and day 28 following vaccination. The study was approved by the Ethics Review Committee of the National Institute for Viral Disease Control and Prevention (NIVDC, assurance number, 200916), and written informed consent was obtained from all volunteers.

### Mice and immunization

*Ifitm3^-/-^* mice were generated using TALENs (transcription activator-like effector nucleases) technology based on a C57BL/6J background mice by Biocytogen (Biocytogen, Beijing) [17] and bred in a pathogen free animal facility at the Chinese Center for Disease Control and Prevention. Seven to ten-week-old female wild type (WT) and congenic *Ifitm3^-/-^* mice were immunized intraperitoneally with 50 μL of TIV (including A/Michigan/45/2015, A/Hong Kong/4801/2014 and B/Victoria/60/2008 viruses) for northern hemisphere 2017–2018 influenza season (lot#201706009) (1.5 μg of each haemagglutinin [HA]) from SINOVAC BIOTECH (SINOVAC, Beijing) followed by a second dose after 14 days.

The animal study was approved by the Animal Ethics Review Committee of the NIVDC (assurance number, 20161125030). Dissections and orbital blood collections were performed under anaesthesia that was induced and maintained with isoflurane. All efforts were made to minimize suffering to the mice.

### Genotyping of *IFITM3* rs12252

The Sequencing and genotyping of *IFITM3* rs12252 were operated following the method described previously [18].

### Haemagglutination inhibition (HI) test

HI assays were performed against the influenza vaccine strains according to the standardized protocol by the World Health Organization [19].

### Enzyme-linked immunosorbent assay (ELISA)

Total IgG against H1N1, H3N2, and B/Victoria viruses were determined by ELISA using split antigens as described previously [20].

### Flow cytometry

Single cell suspensions of murine splenocytes were prepared according to a protocol described previously using cell strainers (Biologix, USA) [21]. Splenocytes were harvested and stained using various combinations of the special Abs: anti-CD3-PE-Cy7(clone 17A2; Biolegend, CA), anti-CD45R/B220-APC-Cy7 (clone RA3-6B2), anti-CD138-PE (clone 281-2), anti-CD38-FITC (clone 90), anti-GL7-Pacific Blue™ (clone GL7), anti-Fas/CD95-APC (clone SA367H8), anti-IgG-PE/Dazzle™ 594 (clone Poly4053). 7-aminoactinomycin D (7AAD) (Biolegend) was also included to exclude dead cells.. Data was analyzed on a FACS Aria^TM^ IIu (BD Biosciences, USA).

### Enzyme-linked immunospot assay (ELISPOT)

TIV-special IgG^+^ antibody secreting cells (ASC) from mice were analyzed by ELISPOT assay on a polyvinylidene difluoride (PVDF) 96-well filtration plate (Millipore, MA) following the manufacturer’s instructions for the Mouse IgG ELISpot^BASIC^ kit (Mabtech, Sweden). The spots were read with an EliSpot Reader System ELRIFL04 (AID, Germany).

### Western blotting (WB)

Lymphocytes were lysed in RIPA buffer (50 mM Tris, 150 mM NaCl, 1 mM EDTA, 1 mM EGTA, 0.1% SDS, 0.5% DOC, and 1% Triton X-100), sonicated, boiled and used for SDS – PAGE followed by Western blotting. The following primary polyclonal antibodies were used: rabbit anti-IFITM3 (Proteintech, Chicago), rabbit anti-BCL-6 (Cell Signaling Technology, MA), rabbit anti-Blimp1 (Cell Signaling Technology), rabbit anti-AID (Cell Signaling Technology) and mouse anti-β-actin (Cell Signaling Technology). Goat anti-rabbit IgG-HRP and rabbit anti-mouse IgG-HRP (Santa Cruz Biotechology, Texas) were used as secondary antibodies. ECL western blotting detection reagents (Solarbio, Beijing) were used for detection.

### Quantitative real-time PCR

Total mouse RNA was extracted and reverse transcribed to cDNA, then quantified with SYBR® Premix Ex Taq (Tli RNaseH Plus) ROX plus kit (Takara, Japan). Samples were assayed in triplicate. Ratios of 2^−ΔΔC(t)^ were computed and analyzed with glyceraldehyde-phosphate dehydrogenase (GAPDH) as the reference gene. The special primers were designed by Primer 5 with sequences downloaded from NCBI (Table S1).

### Statistical analysis of data

Data were analyzed using SPSS (Version 18.0; SPSS Inc., USA). Pearson’s Chi-square test was used to assess the association between SCR and *IFITM3* rs12252 genotypes. The two-tailed Student’s *t*-test or Mann–Whitney test was applied to evaluate the differences between the numbers of various immune cells at different days. *P* values <0.05 were considered statistically significant (* indicates *P *< 0.05, ** indicates *P *< 0.01, *** indicates *P *< 0.001). Graphs were prepared with GraphPad Prism v 5 (GraphPad Software, CA).

## Results

### Antibody responses in adults after TIV immunization

**Figure 1.**
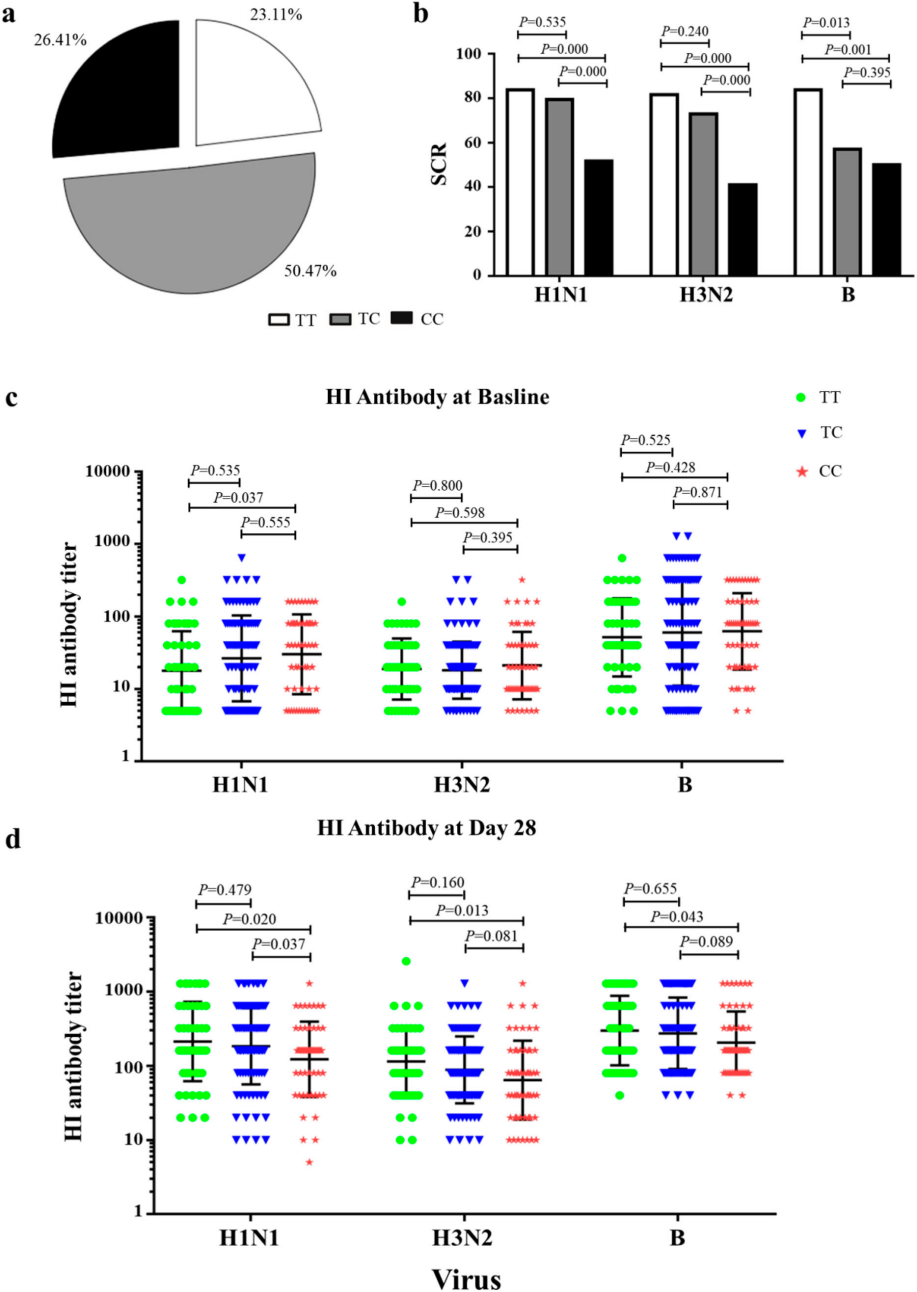
Comparing the SCR among *IFITM3* rs12252-CC, CT, and TT genotypes carriers after TIV vaccination from 2009 to 2015. (a) Distribution about *IFITM3* rs12252-CC, CT, and TT in enrolled 212 volunteers. (b) The SCRs to H1N1, H3N2 and B/Victoria in donors with rs12252-CC, CT, and TT genotypes. (c, d) The HI antibody anti-H1N1, H3N2 and B virus were detected at baseline before influenza vaccination (c) and at day 28 after influenza immunization (d).

Two hundred and twelve healthy adults (age from 18 to 59 years) were enrolled in the study and each received a single dose of seasonal TIV. Whole-blood specimens were collected for HI antibody test and *IFITM3* rs12252 genotyping on days 0 and 28 after immunization. In all, 49 donors (23.11%) demonstrated the *IFITM3* rs12252-T/T genotype, 107 (50.47%) had the *IFITM3* rs12252-C/T genotype, and 56 (26.42%) exhibited the *IFITM3* rs12252-C/C genotype (Figure 1a). Moreover, no significant difference was observed between the three *IFITM3* rs12252 genotypes in different age groups and sexes (Table S2).

HI antibody was measured for each of the three influenza strains. Seroconversion (SC) was defined as the percentage of subjects with either a pre-vaccination HI titre ≦1:10 and a post-vaccination HI titre ≧1:40 or a pre-vaccination HI titre ≧1:10 and a minimum four-fold rise in post-vaccination HI titre [22,23]. Interestingly, the SCR for H1N1, H3N2 and B viruses in donors with *IFITM3* rs12252-C/C genotype was lower compared with donors carrying the T/T genotypes (Figure 1b). The geometric mean titre (GMT) of HI titre against H1N1 virus in donors with IFITM3 rs12252-C/C genotype was higher than that in donors with T/T genotype before TIV immunization (Figure 1c). However, the GMT of HI titre against H1N1, H3N2 and B viruses in donors with IFITM3 rs12252-C/C genotype were lower than donors with TT genotype after TIV immunization (Figure 1d). To explore this interesting phenomenon, we established an IFITM3 deletion mouse model for further investigation.

### IFITM3 deletion resulted in lower antibody response after TIV immunization in mice

**Figure 2.**
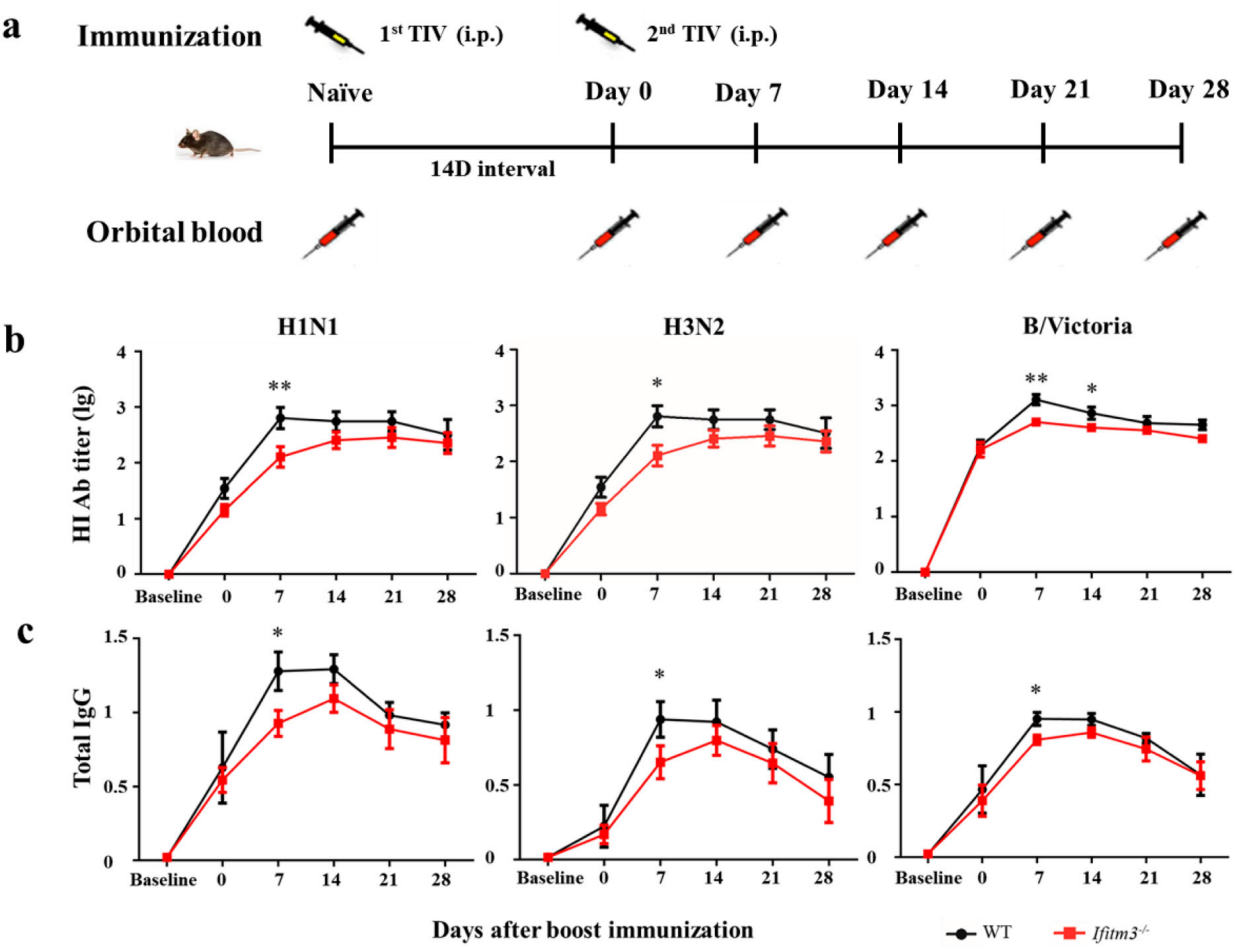
IFITM3 deletion led to low antibody response after TIV immunization in mice. (a) WT and congenic *Ifitm3^-/-^* C57/6 mice (*n* = 5 per group) were immunized twice by intraperitoneal injection of 50 μL of TIV (1.5 μg of each HA). The interval time between two immunizations was 14 days. (b) The serum HI titres were detected by HI against A/Michigan/45/2015 (H1N1), A/Hong Kong/4801/2015 (H3N2) and B/Brisbane/60/2008 (B/Victoria). (c) Total IgG was investigated by ELISA with split H1N1, H3N2 and B/Victoria viruses (1 μg/ml of each HA). The y-axis represents OD_450nm/630nm_. The x-axis shows days after boost immunizations. The bars represent the mean values and the standard errors of the means. Significant differences are marked by * *P *< 0.05, ** *P *< 0.01, *** *P *< 0.001.

To investigate the impact of IFITM3 on the antibody response, we established a model of *Ifitm3^-/-^* mice and continuously measured the antibody level for a month after inoculating WT and *Ifitm3^-/-^* mice via the intraperitoneal route with TIV. We collected serum from orbital blood before immunization and at days 0 (14 days after primary immunization), 7, 14, 21 and 28 after booster immunization and detected the HI titre and IgG against H1N1, H3N2 and B/Victoria viruses (Figure 2a). The peaks of HI titre against H1N1 and H3N2 viruses occurred at day 14 in *Ifitm3^-/-^* mice, which were later compared with those in WT mice. The HI titre against H1N1, H3N2 and B/Victoria viruses in *Ifitm3^-/-^* mice were lower than those in WT mice after booster vaccination (Figure 2b). Especially at day 7 after booster immunization, the GMT of HI titre against H1N1, H3N2 and B/Victoria viruses were 3.49, 5.01 and 2.20-fold higher, respectively, in WT mice than those in *Ifitm3^-/-^* mice (Table S3).

Simultaneously, *Ifitm3^-/-^* mice showed decreased production of TIV-specific IgG after TIV immunization compared with WT mice (Figure 2c). The levels of total IgG peaked at day 14 in *Ifitm3^-/-^* mice, which was later than the peak observed in WT mice, for which the peak total IgG was at day 7. These results showed that *Ifitm3^-/-^* mice demonstrated slow production of antibody and induced a weaker antibody response compared with WT mice after TIV immunization.

### IFITM3 deletion reduced the number of activated GC B cells in spleens after TIV immunization

**Figure 3.**
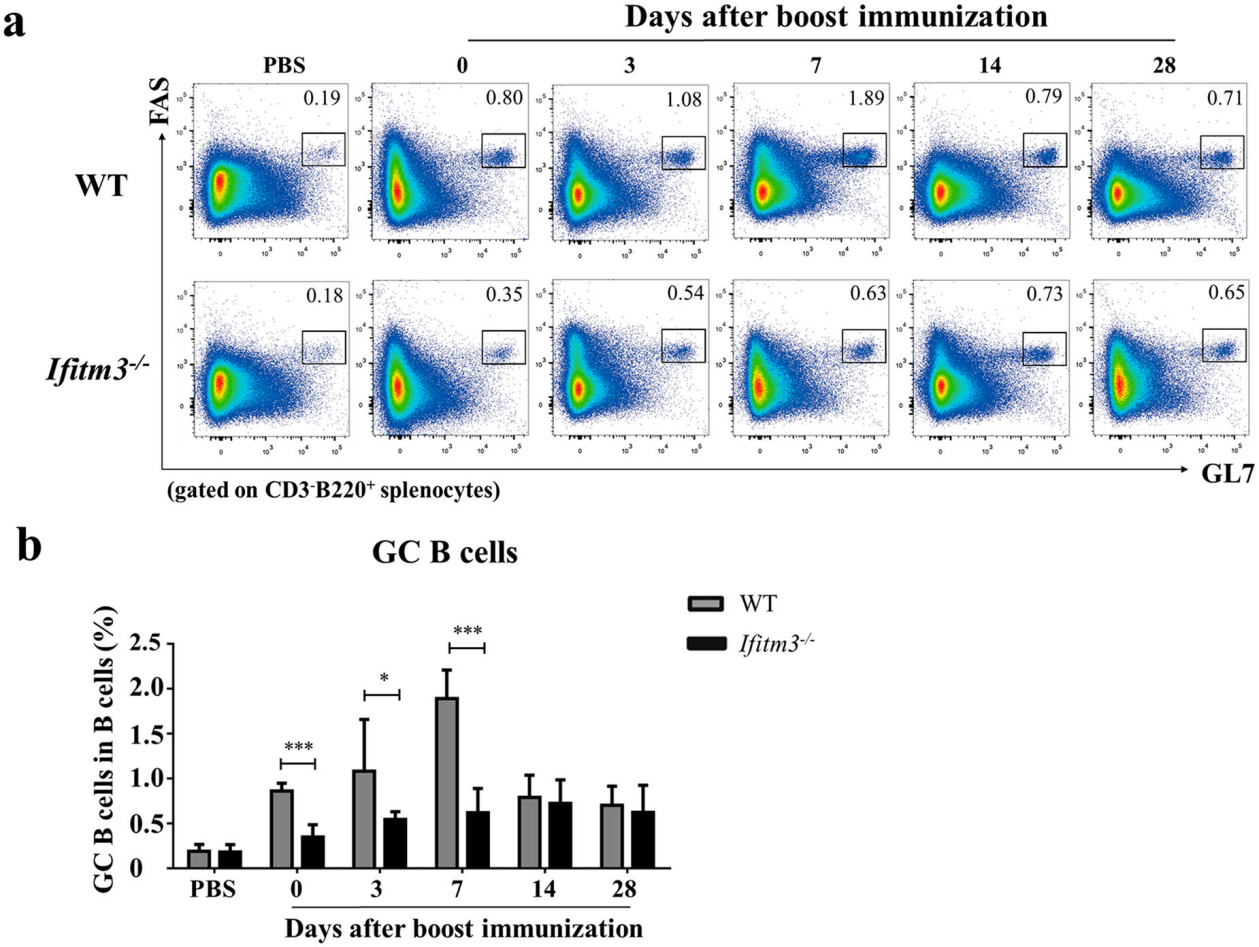
IFITM3 deletion reduced the number of activated GC B cells in spleens after TIV immunization. (a) Lymphocytes were isolated from splenocytes from each group of mice. The populations of CD3^-^B220^+^FAS^+^GL7^hi^ cells were defined as GC B cells and analyzed by flow cytometric analysis. (b) The percentage of GC B cells among B220^+^ B cells were compared between WT and *Ifitm3^-/-^* mice at days 0, 3, 7, 14 and 28 after booster immunization (*n* = 5 per group). Significant differences are marked by * *P *< 0.05, ** *P *< 0.01, *** *P *< 0.001.

Having demonstrated the low antibody response in *Ifitm3^-/-^*mice after TIV immunization, we sought to address the variation in B cells which secrete antibody. The antibody response to T cell-dependent antigen is depended on GCs. Within GCs, B cells activate and proliferate to become antibody-secreting plasma cells and memory B cells [24,25]. Thus, we first analyzed splenic GC B cells by flow cytometry at days 0, 3, 7, 14 and 28 after booster immunization (Fig. S1; Figure 3a). In control mice immunized with PBS, there was no difference between the numbers of GC B cells in spleens of WT and *Ifitm3^-/-^* mice. However, we observed a decrease in the frequencies of splenic GC B cells in *Ifitm3^-/-^* mice at days 0, 3 and 7 after boosting (Figure 3b). The overall numbers of GC B cells peaked at day 7 in WT mice, but was delayed until at day 14 in *Ifitm3^-/-^* mice. Furthermore, the number of GC B cells was three-fold higher in WT mice than in *Ifitm3^-/-^* mice at day 7 after boosting. These data show that IFITM3 deletion decrease the number of splenic GC B cells and postpone the peak of activated GC B cells.

### Dysfunction of GC-to-plasma/memory transition was detected in *Ifitm3^-/-^* mice

**Figure 4.**
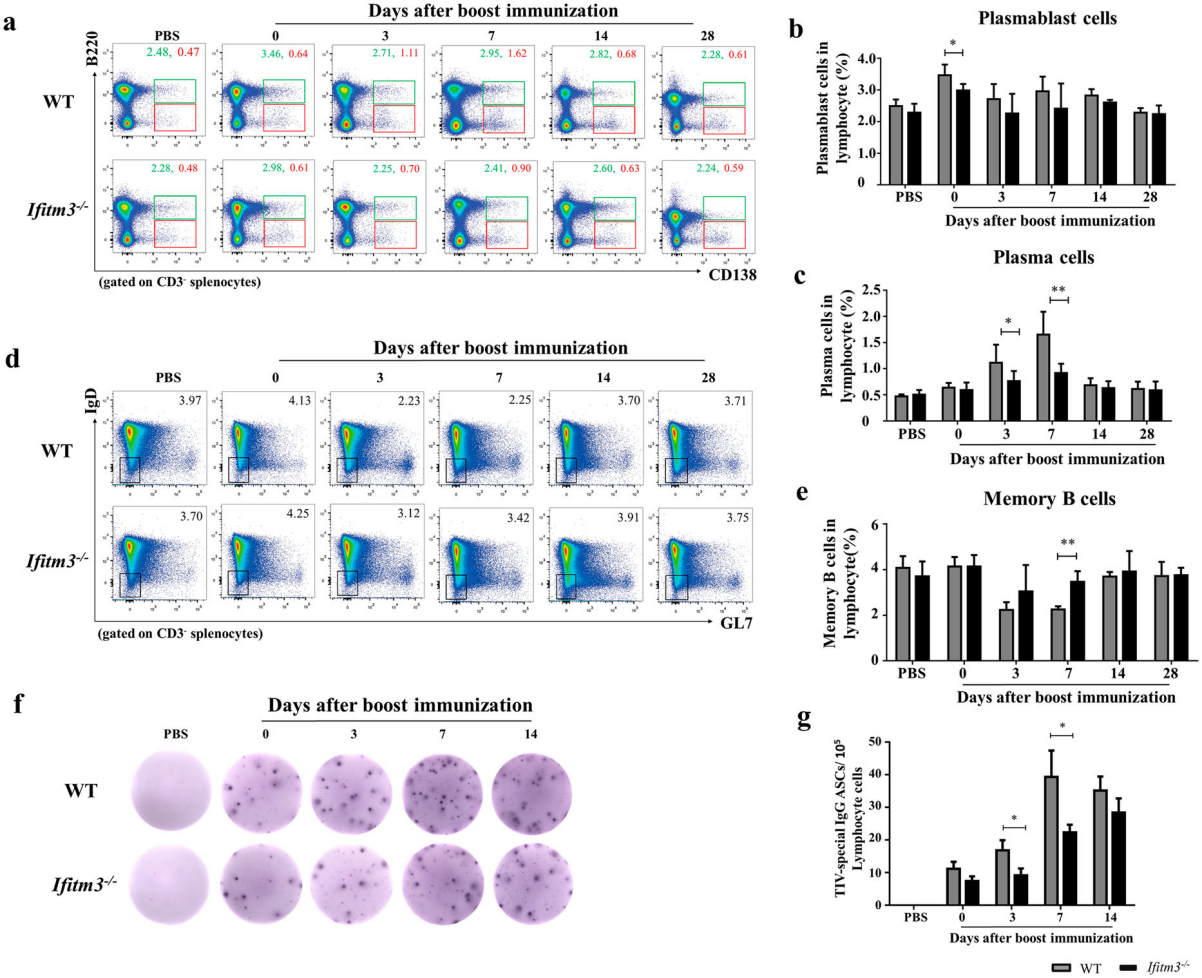
The dysfunction of plasma and memory cells differentiation in *Ifitm3^-/-^* mice. (a) The population of plasmablasts were defined as CD3^-^B220^+^CD138^+^ and gated with green rectangle by flow cytometric analysis. The population of plasma cells were confirmed as CD3^-^B220^low^CD138^+^ and gated with red rectangle. (b, c) The percentage of plasmablasts and plasma cells among lymphocytes were respectively analyzed between WT and *Ifitm3^-/-^* mice at days 0, 3, 7, 14 and 28 after TIV boost immunization (*n *= 5 per group). (d) The phenotype of memory cells were defined as CD3^-^B220^+^CD138^-^GL7^−^IgD^−^ by flow cytometry. (e) The proportion of memory cells among lymphocytes was analyzed between WT and *Ifitm3^-/-^* mice at days 0, 3, 7, 14 and 28 after boost immunization (*n* = 5 per group). (f) TIV-specific IgG^+^ ASCs were assayed by an ELISPOT with the lymphocytes in the spleens of WT and *Ifitm3^-/-^* mice at days 0, 3, 7 and 14 after booster immunization. (g) The histogram shows the representative spots at days 0, 3, 7 and 14 after booster (*n* = 5 per group). The bars represent the mean values and the standard errors of the means. Significant differences are marked by * *P *< 0.05, ** *P *< 0.01, *** *P *< 0.001.

In GC, primed B cells differentiate into plasmablasts, plasma cells and memory B cells for secretion of antibody. Antigen-specific plasma cells and memory B cells appear within 1 week and peak in the peripheral blood around day 7 post-influenza immunization, which indicates that the GC reaction is remarkably efficient [26]. We also compared the numbers of plasmablasts, plasma- and memory B cells in spleens of WT and *Ifitm3^-/-^* mice (Fig. S2; Figure 4a-e). The number of plasmablasts was decreased in *Ifitm3^-/-^* mice compared with WT mice, though there was a significant difference at day 0 after booster immunization only (Figure 4b). The number of plasma cells in *Ifitm3^-/-^* mice was substantially reduced compared with WT mice at days 3 and 7 after boosting (Figure 4c). In contrast, we observed that the number of memory B cells was higher in spleens of *Ifitm3^-/-^* mice compared with WT mice at day 7 after booster immunization (Figure 4e).

We further analyzed the numbers of TIV-specific IgG^+^ ASCs by ELISPOT assay at days 0, 3, 7 and 14 after booster immunization (Figure 4f). As expected, the numbers of TIV-specific IgG^+^ ASCs in WT mice were two-fold higher than those in *Ifitm3^-/-^* mice at days 3 and 7 (Figure 4g). Thus, these results indicate that IFITM3 deletion reduces the number of TIV-specific IgG^+^ ASCs, but the number of memory cells in spleens of *Ifitm3^-/-^* mice increases.

### Follicular helper T (T_FH_) cells reduced in *Ifitm3^-/-^* mice

**Figure 5.**
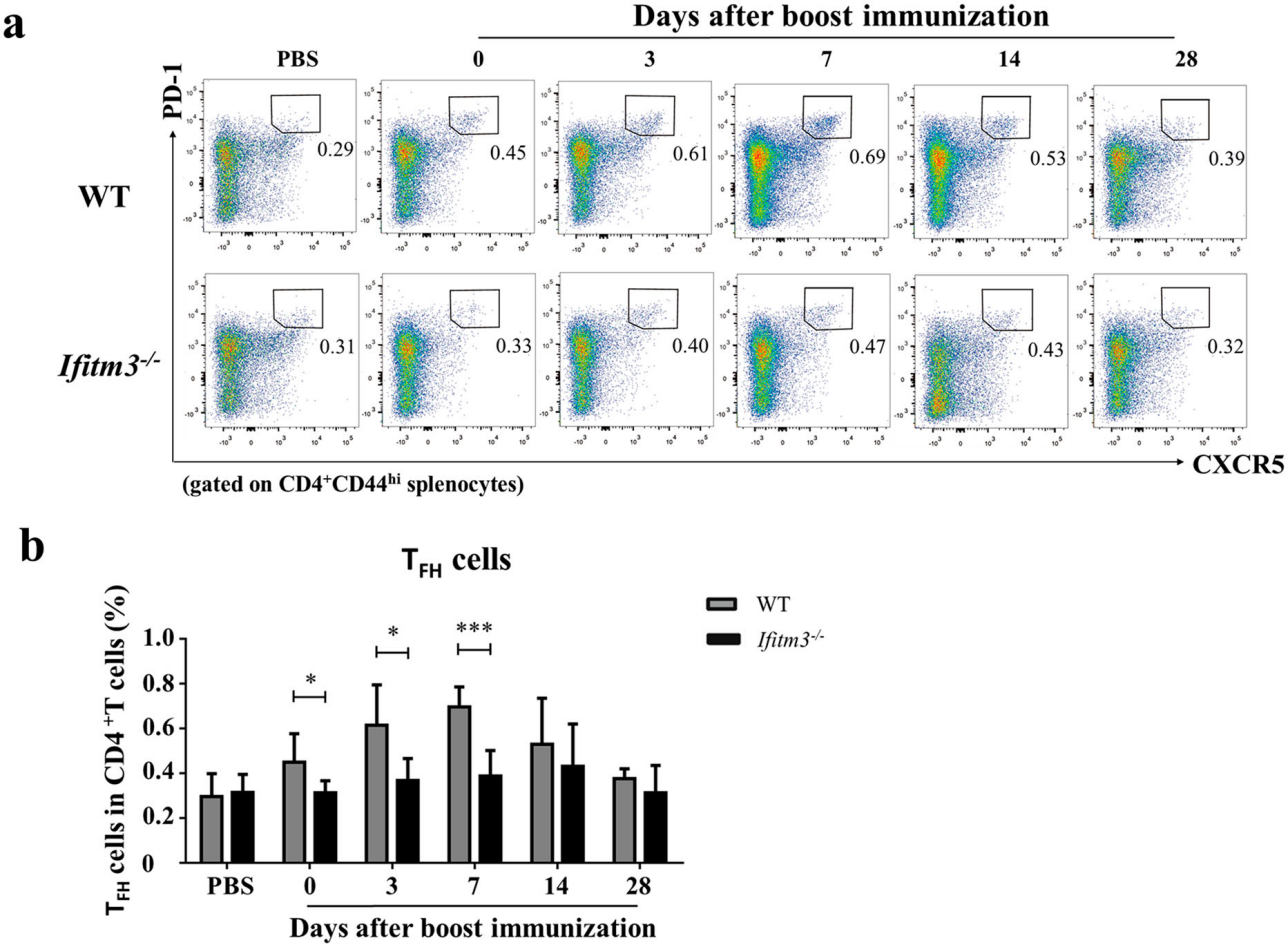
The numbers of T_FH_ cells were reduced in spleens of *Ifitm3^-/-^* mice after TIV immunization. (a) Lymphocytes were isolated from splenocytes from each group of mice. The populations of CD3^+^CD4^+^CD44^hi^CXCR5^hi^PD1^hi^ cells were defined as T_FH_ cells and analyzed by flow cytometric analysis. (b) The percentage of T_FH_ cells among CD3^+^CD4^+^ T cells were compared between WT and *Ifitm3^-/-^* mice at days 0, 3, 7, 14 and 28 after booster (*n* = 5 per group). The bars represent the mean values and the standard errors of the means. Significant differences are marked by * *P *< 0.05, ** *P *< 0.01, *** *P *< 0.001.

Inside GCs, T_FH_ cells provide critical cellular interactions and cross-signalling to antigen-experienced B cells to undergo proliferation, isotype switching, and somatic hypermutation (SHM) in order to generate long-lived plasma cells and memory B cells [27,28]. IFITM3 deletion reduced the antibody level and numbers of splenic GC B cells. So how then do the numbers of T_FH_ cells which are required for GC formation vary between WT and *Ifitm3^-/-^* mice? We analyzed the numbers of T_FH_ cells from spleens of *Ifitm3^-/-^* mice and found them to decline at days 0, 3 and 7 after booster immunization (Fig. S2; Figure 5a, b). The variations of T_FH_ cells were similar to the trends observed for GC B cells in WT and *Ifitm3^-/-^* mice. The overall numbers of T_FH_ cells peaked at day 7 in WT mice, but no peak was observed in *Ifitm3^-/-^* mice. Furthermore, the number of T_FH_ cells was two-fold higher in WT mice than in *Ifitm3^-/-^* mice on day 7 after booster. Collectively, our results further show that a reduction of T_FH_ cells occurs in spleens of *Ifitm3^-/-^* mice.

### Ab levels in *Ifitm3^-/-^* mice remained lower than those in WT mice after third TIV immunization

**Figure 6.**
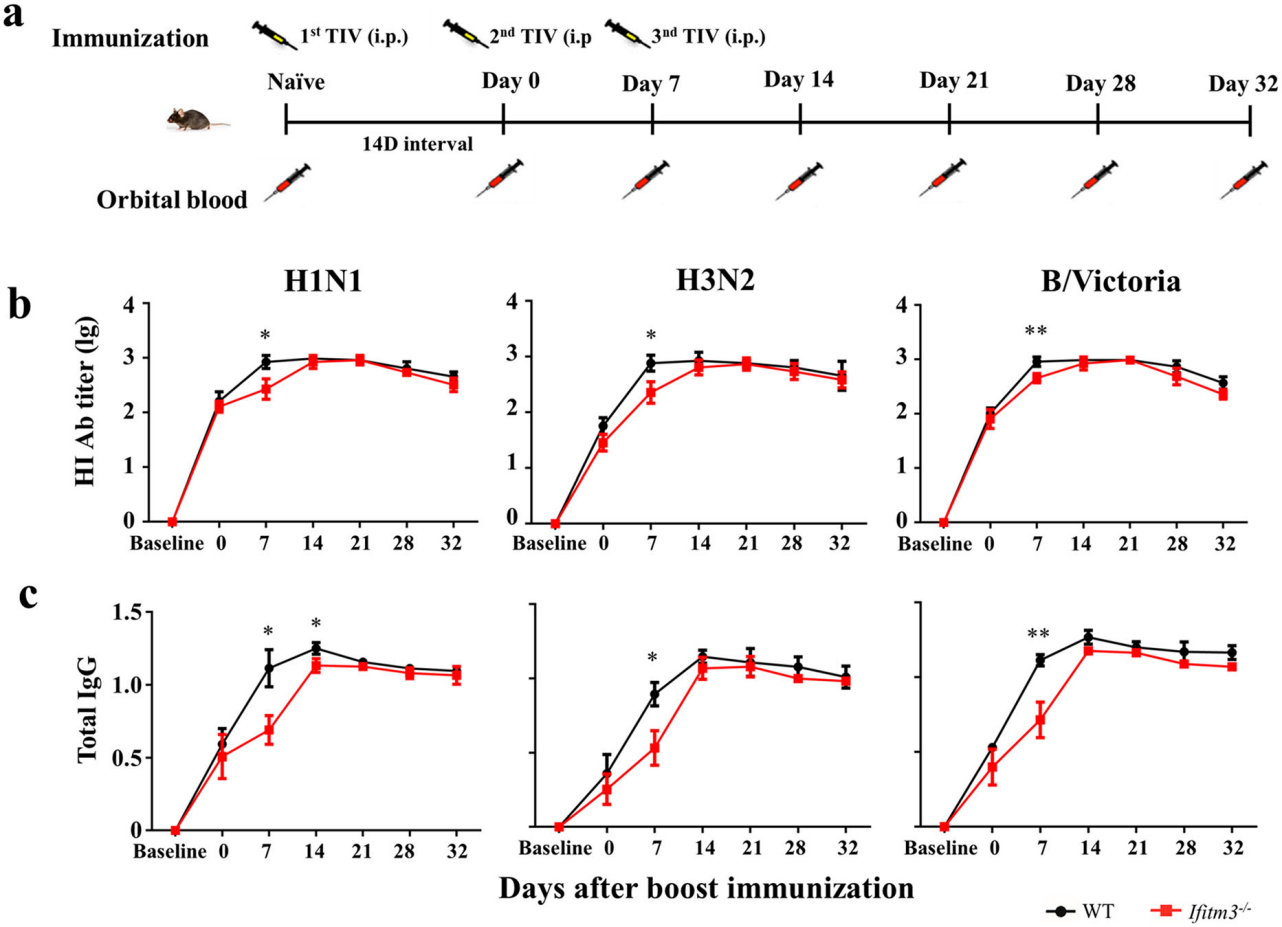
IFITM3 deletion led to low antibody response after third TIV immunization in mice. (a) WT and congenic *Ifitm3^-/-^* C57/6 mice (*n* = 5 per group) were immunized third by intraperitoneal injection of 50 μL of TIV (1.5 μg of each HA). The twice immunization was at day 14 after primary immunization, then the third immunization was at day 7 after twice immunization (*n* = 5 per group). (b) The serum HI titres were detected by HI against A/Michigan/45/2015 (H1N1), A/Hong Kong/4801/2015 (H3N2) and B/Brisbane/60/2008 (B/Victoria). (c) Total IgG was investigated by ELISA with split H1N1, H3N2 and B/Victoria (1 μg/ml of each HA). The y-axis represents OD_450nm/630nm_. The x-axis shows days after boost immunizations. The bars represent the mean values and the standard errors of the means. Significant differences are marked by * *P *< 0.05, ** *P *< 0.01, *** *P *< 0.001.

To verify if the increased numbers of memory B cells resulted in a more powerful antibody response in *Ifitm3^-/-^* mice after TIV immunization, we detected the antibody level after a third TIV vaccination (Figure 6a). The third immunization was implemented at day 7 after the booster vaccination. HI and IgG were detected at days 0 (for twice vaccination), 7 (for third vaccination), 14, 21, 28 and 32. The HI and IgG levels were lower in *Ifitm3^-/-^* mice at day 7 after boosting compared with WT mice, then strongly increased at day 14 (day 7 after third boosting) (Figure 6b, c). Despite this, the HI and IgG level of *Ifitm3^-/-^* mice remained lower than WT mice and there was no significant difference between WT and *Ifitm3^-/-^* mice.

### Transcriptional network regulating GC B cells and plasma cells differentiation was abnormal in *Ifitm3^-/-^* mice

**Figure 7.**
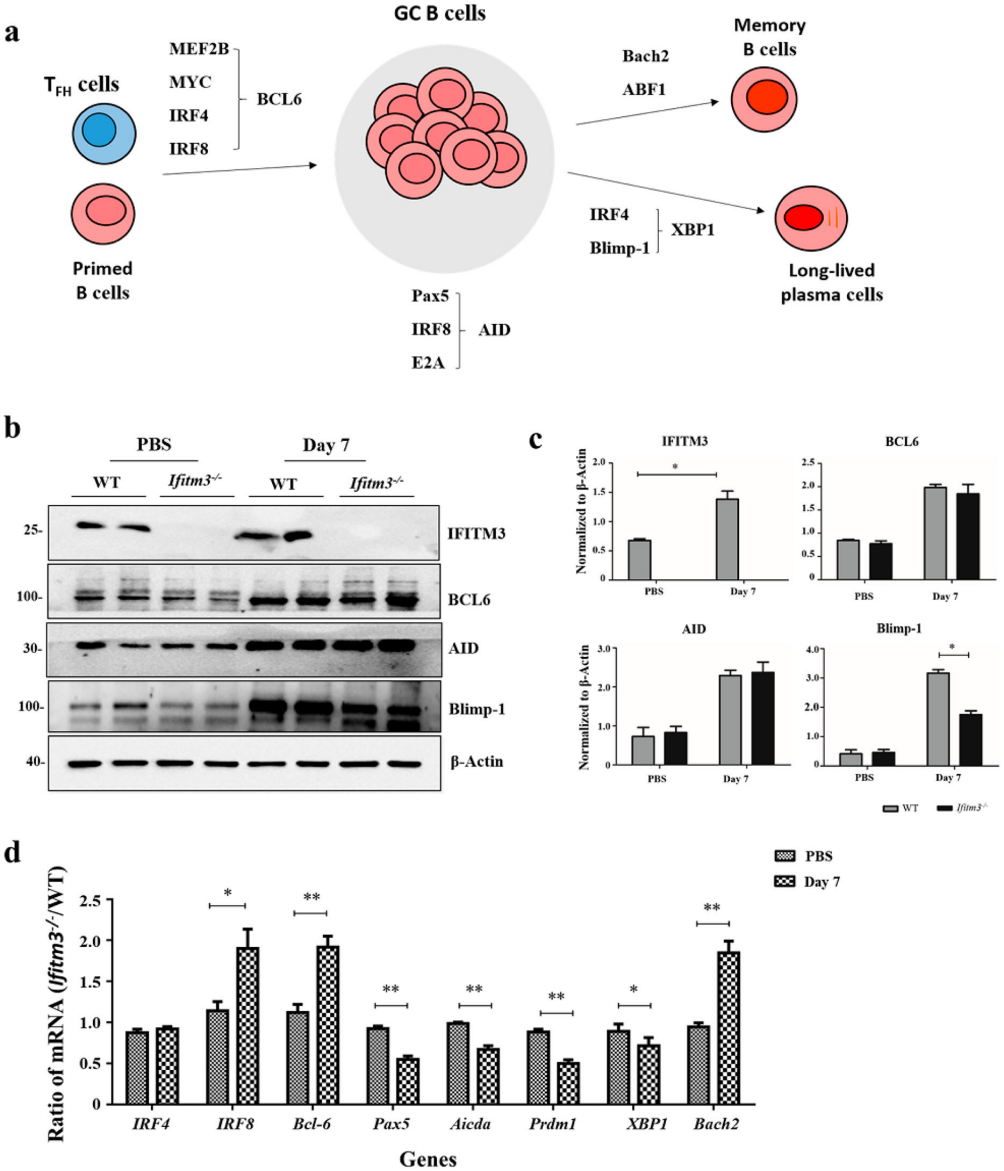
Transcriptional networks regulating GC B cells and plasma cells differentiation were detected by WB and qRT-PCR. (a) The transcription factors controlling GCs formation. * Primed B cells mean Ag-specific naive or memory B cells. (b) WB with anti- IFITM3, anti- BCL6, anti- AID, anti- Blimp-1 and anti- β-Actin revealing the difference between WT and *Ifitm3^-/-^* mice. (c) Quantitative results of the ratios of IFITM3, BCL6, AID, Blimp-1 to β-Actin are showed. (d) The mRNA profiles were analyzed using the 2^−ΔΔCt^ method and results were plotted as fold differences of relative expression normalized to *GAPDH* and ratio of *Ifitm3^-/-^* to WT individually (*n* = 5 per group). The bars represent the mean values and the standard errors of the means. * *P *< 0.05, ** *P *< 0.01.

The transcription factors involved during proliferation and differentiation of GC B cells into plasma cells is well characterized (Figure 7a) [29,30]. T cells are committed to become T_FH_ cells, which upregulate B cell lymphoma 6 (BCL6). BCL6 is a master regulator which progresses primed B cells to upregulate BCL6 at both the RNA and protein levels and further differentiate into GC B cells [31]. Proliferating GC B cells characteristically have large amounts of activation-induced cytidine deaminase (AID), encoded by the *Aicda* gene and is the enzyme responsible for SHM and class switch recombination which generate a wide repertoire of affinities for specific antibody [32,33]. GC B cells differentiate into plasma cells which need large quantities of B lymphocyte–induced maturation protein 1 (Blimp-1), encoded by the *Prdm1* gene [34]. Blimp-1, together with IRF4, acts upstream of X-box binding protein 1 (XBP1), a transcription factor that is essential for upregulation of the secretory apparatus required for antibody production in plasma cells [35,36]. Bach2 is as a factor in the promotion of the entry of light-zone GC cells into the memory B cell compartment [37].

We analyzed the expression of the transcription factors by WB and the corresponding abundance of mRNAs by quantitative PCR using splenic lymphocytes at day 7 after booster. Firstly, the expression of IFIMT3 was substantially increased in WT mice after immunization which is consistent with the previous research (Figure 7b, c) [38]. We also observed that *Ifitm3^-/-^* mice showed a substantial reduction in the expression of Blimp-1 protein in spleens at day 7, whereas the expressions of BCL6 and AID were similar when WT mice were compared with *Ifitm3^-/-^* mice (Figure 7b, c). Additionally, there were substantial decreases in *Pax5*, *Aicda*, *Prdm1* and *XBP1* mRNA in the splenic lymphocytes of *Ifitm3^-/-^* mice at the same time point. However, the *Bcl6, IRF8* and *Bach2* mRNA were substantially increased in spleen of *Ifitm3^-/-^* mice at days 7 after boosting (Figure 7d). These results indicate that the expression of Blimp-1 declines at day 7 after booster immunization while at the mRNA level, *Bcl6* and *Bach2* mRNA increase in *Ifitm3^-/-^* mice at this time.

## Discussion

IFITM3 is an important antiviral factor in restricting influenza virus infection. The SNP rs12252-C allele of *IFITM3* had been linked with severe illness in adults during pandemic influenza H1N1/09 virus infections, but the mechanisms remain incompletely understood. We were curious about whether IFITM3 plays an important role in the regulation of the immune system and so we investigated the antibody responses of three IFITM3 rs12252 genotype carriers after TIV immunization. Our results showed that *IFITM3* rs12252-C/C carriers exhibited a lower level of antibody response to H1N1, H3N2 and B viruses compared with rs12252-T/T carriers after TIV immunization, suggesting that the *IFITM3* genotype may be associated with antibody response. The same phenomenon was also observed in *Ifitm3^-/-^* mice after TIV immunization. These findings reveal a previously unappreciated role for IFITM3 in the antibody response. The research from *Ling Qin et al* who investigated the influenza vaccination response is associated with IFITM3 gene in human population. The results showed that the median fold of increasing HI antibody to pdm09H1N1 in volunteers with the rs12252-C/C genotype was much lower than the change in rs12252-T/T genotypes on both days 14 and 28 after vaccination [15]. These results supported our conclusions as well.

GCs are the principal sites of affinity maturation of antigen-specific immunoglobulin, transient structures induced by T cell-dependent (TD) antigens [39]. T_FH_ cells orchestrate the GC response in part by providing help factors for which GC B cells compete to survive and undergo clonal expansion [40]. Proliferating GC B cells differentiate into plasma cells to produce antibody, and simultaneously differentiate to memory cells which produce powerful antibody responses. The numbers of GC B and plasma cells and TIV-specific IgG^+^ ASCs were decreased in *Ifitm3^-/-^* mice, which may explain the lower level of antibody response. We also found a decreased number of GC T_FH_ cells in *Ifitm3^-/-^* mice. Furthermore, the expression levels of some factors of the transcriptional network regulating the cellular dynamics of splenic GC B cells, high-affinity antibody development and plasma cell differentiation, such as BCL6 or Blimp1, which are considered to be master regulators for GC or plasma cell development, were abnormal in *Ifitm3^-/-^* mice [32]. BCL6 inhibits the transcription of Prdm1, the gene that encodes Blimp-1, and Blimp-1 represses transcription of the genes that encode BCL6 [34]. So, BCL6 is a known inhibitor of plasma cell differentiation and thus promotes memory B cell differentiation [41,42]. In this study, the increased *Bcl6* mRNA and decreased expression of Blimp-1 and *Prdm1* mRNA may be a reasonable explanation for the dynamic cells observed within GCs in *Ifitm3^-/-^* mice. As expected, IFITM3 deletion resulted in diminished numbers of plasma cells and increased the memory cells. A previous study reported that IFITM3 loci contain several potential BCL6 binding sites and BCL6 could bind to these sites, including the promoter and 3’ conserved untranslated region to inhibit the expression of MX2 and IFITM3 in T_FH_ cells by chromatin immunoprecipitation assay [43]. Our findings indicate that BCL6 may have a close connection to IFITM3, giving us a hypothesis that IFITM3 deletion results in the high expression of BCL6 in T_FH_ cells or other immune cells and then impacts on the antibody response. However, the mechanisms involved require further investigation.

Our results show that IFITM3 deletion diminishes the antibody response and *IFITM3* SNP rs12252-C/C carriers show lower antibody levels after TIV immunization. The frequency of the *IFITM3* SNP rs12252-C is much higher in the Han Chinese population than in Caucasians [18]. A comprehensive knowledge of influenza-IFITM3 interactions is necessary to develop a novel and alternative anti-influenza virus strategy in carriers of the *IFITM3* SNP rs12252-C/C. *IFITM3* SNP rs12252-C could be used to identify patients at high risk of severe illness, which would be of immense benefit for prioritizing treatment.
